# Mn-Doped CeO_2_ Nanozyme-Integrated Mesoporous Interfaces for High-Sensitivity Antifouling Electrochemiluminescence Biosensing

**DOI:** 10.3390/bios15070411

**Published:** 2025-06-27

**Authors:** Guanze Huang, Haiyan Qiu, Huiping Chen, Wanxuan Li, Yufei Zhang, Minfang Huang, Tingting Zhang, Xiaoxin Xu, Shanwen Hu

**Affiliations:** 1 Department of Health Inspection and Quarantine, School of Public Health, Fujian Medical University, Fuzhou 350122, China; hgz000506@fjmu.edu.cn (G.H.); 2220210169@fjmu.edu.cn (H.Q.); chp2240210259@fjmu.edu.cn (H.C.); liwanxuan@stu.fjmu.edu.cn (W.L.); zhangyufei@stu.fjmu.edu.cn (Y.Z.); shanwenhu@fjmu.edu.cn (S.H.); 2 Jiangsu Key Laboratory for Chemistry of Low-Dimensional Materials, School of Chemistry and Chemical Engineering, Huaiyin Normal University, Huaian 223300, China; 3 Key Laboratory for Analytical Science of Food Safety and Biology, Ministry of Education, College of Chemistry, Fuzhou University, Fuzhou 350002, China; 221320071@fzu.edu.cn

**Keywords:** electrochemiluminescence (ECL), nanozyme sensing, anti-fouling coating

## Abstract

To address the challenges of nonspecific adsorption interference and low mass transfer efficiency encountered by electrochemiluminescence (ECL) sensors in complex biological matrices, this study developed a Mn@CeO_2_ nanozyme-based sensing interface. The Mn-doped CeO_2_ enhanced electron transfer efficiency, increased oxygen vacancy concentration, and stabilized the Mn-O-Ce structure, collectively enabling highly efficient peroxidase (POD)-like activity. The design significantly improved ECL reaction efficiency, which simultaneously conferred synergistic antifouling and mass transport enhancing properties. The mesoporous silica nanoparticle on the sensing interface accelerated mass transfer processes, thereby overcoming the limitations of traditional diffusion-controlled kinetics. The Mn@CeO_2_ nanozyme and mesoporous silica nanoparticle synergistically improved electron transfer and reactant enrichment, thereby significantly enhancing the signal response. Concurrently, a biomimetic anti-fouling coating was introduced at the interface to effectively suppress nonspecific adsorption of interferents. The constructed nanozyme-enhanced ECL sensing platform was demonstrated through the detection of dopamine (DA) as a model neurotransmitter, exhibiting favorable detection performance while maintaining high-accuracy detection in complex biological samples. This strategy offers a novel approach to developing highly sensitive and interference-resistant ECL sensors, with promising applications in disease biomarker monitoring and live physiological sample analysis.

## 1. Introduction

Chemiluminescence is a phenomenon where light is directly produced during a chemical reaction without the need for external light sources to excite it. This phenomenon has wide applications in both bioluminescence and artificial chemiluminescence systems [1]. Electrochemiluminescence (ECL) technology, which integrates electrochemical reactions with chemiluminescence, has become an important tool in the field of analytical chemistry due to its advantages of high sensitivity, low background signal, and broad dynamic range [2,3]. However, traditional ECL systems are often constrained by issues such as the self-decomposition of coreactants, high background signals, and poor stability, thereby limiting their application scenarios [4]. In recent years, nanomaterials have been widely used for ECL performance optimization due to their unique electron transfer properties and tunable optoelectronic properties [5,6,7]. In particular, nanomaterials with enzyme-like catalytic activity (nanoenzymes) can significantly enhance the electron transfer efficiency and reduce the overpotential in ECL reactions by mimicking the catalytic mechanism of natural enzymes [8,9]. By integrating the structural advantages of nanomaterials with the high selectivity of enzyme catalysis, these materials can precisely regulate the electron transfer paths at the interface of the ECL reaction, which provides a new strategy for constructing a high signal-to-noise ratio and anti-interference sensing system.

In recent years, the enzyme-like properties of CeO_2_ have attracted considerable attention in ECL applications. The rapid redox cycling of Ce^3+^/Ce^4+^ and the abundant oxygen vacancy structures in CeO_2_ endow it with remarkable performance in ECL signal amplification. However, despite the tunable physicochemical properties, controllable size, morphology, and surface states of CeO_2_ catalysts synthesized through various methods, which enable different catalytic behaviors under different conditions, the catalytic activity and specificity of CeO_2_ in physiological environments remain lower than those of natural enzymes [10]. To address this issue, researchers have proposed metal/metal oxide composite strategies. Combining CeO_2_ with metals or metal oxides generates synergistic effects, which enhance electron transfer, increase surface active sites, and regulate oxygen vacancy concentrations [11]. For instance, Pt, known for its excellent electrocatalytic activity, can effectively promote the reduction of H_2_O_2_. In the Pt/CeO_2_/NCNFs composite synthesized by Zhan, Pt nanoparticles dispersed around CeO_2_ nanosheets not only enhanced the electrocatalytic activity but also, in conjunction with the CeO_2_ nanosheets, further improved the electrochemical performance of the composite material [12]. Pd, as a single-atom catalyst, can be uniformly dispersed in CeO_2_ nanostructures to form Pd–O–Ce bonds, increasing the density of oxygen vacancies and thereby significantly enhancing the catalytic activity of the nanozyme [13]. Moreover, Mn-doped CeO_2_ nanozymes have demonstrated higher peroxidase (POD)-like activity by improving electron transfer efficiency, increasing oxygen vacancy concentration, and stabilizing the Mn-O-Ce structure [14]. Additionally, the introduction of Mn alters the electronic structure of CeO_2_, increasing the content of Ce^3+^ and thereby enhancing electrical conductivity and charge transfer capability of the material. These improvements not only enhance catalytic activity but also contribute to the sensitivity and response speed of electrochemical sensors [15].

Beyond substrate specificity and limited catalytic activity, the anti-interference performance of electrodes is another significant challenge for electrochemical biosensors based on nanozymes. Various anti-fouling strategies have been employed to maintain sensing performance in complex media, and these strategies have proven to be effective [16,17,18]. For example, peptide nanoparticles formed by the self-assembly of anti-fouling peptides have demonstrated excellent stability and anti-fouling properties in preventing the adsorption of non-specific materials such as proteins and cells from biological samples [19]. The copolymer PSN, composed of zwitterionic sulfobetaine methacrylate and hydrophobic N-isopropylacrylamide, exhibits exceptional anti-fouling performance in serum due to its hydrophobic anchoring and zwitterionic polymer anti-fouling properties. The anti-fouling sensors constructed in this manner possess a large specific surface area, good conductivity, and do not require complex operations, providing valuable insights for the design of electrochemical biosensors for fluid samples [20].

Therefore, in response to the challenges of detecting complex samples, the synergistic innovation of confinement effects and anti-fouling strategies has become a key focus in nanozyme research. Herein, we have designed a novel Mn@CeO_2_-based composite nanozyme to synergistically enhance the luminol ECL system. Due to the large specific surface area, high pore volume, and large pore size of mesoporous SiO_2_, we introduced star-shaped mesoporous nano-silica to provide abundant adsorption sites for luminol molecules, thus overcoming the limitations of traditional diffusion kinetics [21]. This design significantly shortens the electron transfer distance between reactive oxygen species (ROS) and luminol-derived radicals, thereby enhancing the luminescence efficiency of the luminol system. Furthermore, we have applied phase-transited bovine serum albumin (PTB) as an anti-fouling coating to prevent non-specific molecular interference during the detection of complex samples and to extend the applicability of the biosensor to complex biological matrices. This sensor addresses the shortcomings of traditional ECL detection methods, such as low luminescence efficiency and insufficient anti-interference capability, enabling the detection of dopamine (DA) in complex samples at low concentrations with excellent linear response and high sensitivity. These advancements highlight its potential for broader applications in public health monitoring and early disease screening.

## 2. Materials and Methods

### 2.1. Instrumentations and Materials

Phosphate buffered saline (PBS, pH 7.4–7.6) was purchased from Shanghai Sangon Biotech Company Ltd. (Shanghai, China). Cetyltrimethylammonium chloride (CTAC), triethanolamine (TEA), tetraethyl orthosilicate, potassium ferrocyanide (K_4_[Fe(CN)_6_]), potassium ferricyanide (K_3_[Fe(CN)_6_]), potassium chloride (KCl), sodium hydroxide (NaOH, 96%), dopamine hydrochloride (DA, 98%), and luminol (98%), were purchased from Shanghai Macklin Biochemical Technology Company Ltd. (Shanghai, China). Chitosan (CTS, Deacetylation 95%, 100–200 mpa.s) and bovine serum albumin (BSA, 98%) were purchased from Shanghai Aladdin Biochemical Technology Company Ltd. (Shanghai, China); 3,3′,5,5′-tetramethylbenzidine (TMB) was purchased from Beijing ZOMAN Biotechnology Company Ltd. (Beijing, China). Tris-(2-carboxyethyl)-phosphine hydrochloride (TCEP) was purchased from Sangon Biotech Company Ltd. (Shanghai, China), ethanol (EtOH) was purchased from Beijing Innochem Company Ltd. (Beijing, China). Cerium (III) nitrate hexahydrate (Ce(NO_3_)_3_·6H_2_O, 99%), potassium permanganate (KMnO_4_, 99%), methenamine (HMT, 98%), and hexadecyltrimethylammonium bromide (CTAB, 99%) were purchased from Sigma-Aldrich (St. Louis, MO, USA). Fetal bovine serum (FBS) was purchased from Gibco Life Sciences Company Ltd. (Grand Island, NY, USA). Experimental water was ultrapure water (18.2 MQ cm) prepared using a Milli-Q system.

### 2.2. Preparation of CeO_2_@Mn

Mn@CeO_2_ bimetallic nanoenzymes were synthesized by a hydrothermal method. In this process, Ce(NO_3_)_3_·6H_2_O (0.1 M), hexamethylenetetramine (HMT, 0.1 M), and CTAB (0.1 M) were dissolved in 10 mL of ultrapure water, then reacted at 80 °C for 5 h to form the CeO_2_ precursor solution. In situ loading of Mn_3_O_4_ onto the CeO_2_ surface was then achieved by continuing the reaction at 80 °C for 5 h with potassium permanganate (KMnO_4_, 0.1 M) as the Mn source. After the reaction, the supernatant was removed by centrifugation at 1500× *g* for 15 min, and the product was washed sequentially with ultrapure water and ethanol three times, then dried under vacuum at 60 °C for 12 h to obtain the Mn@CeO_2_ composite nanoenzyme.

### 2.3. Preparation of the SiO_2_

The SiO_2_ was synthesized using modified previous studies based on an oil-water biphase reaction. In brief, 24 mL of CTAC (25 wt%) and 0.18 g of TEA were added to 36 mL of water and stirred gently at 60 °C for 1 h in a 100 mL round-bottom flask. Then, 20 mL of (2.5% *v*/*v*) tetraethyl orthosilicate in cyclohexane was carefully added to the CTAC-TEA solution and maintained at 60 °C for 18 h. Afterward, the product was washed with ethanol (EtOH) and water three times by centrifugation at 18,000× *g* for 15 min and finally dried [22].

### 2.4. Preparation of the CTS

The 1% chitosan solution is prepared with 1% acetic acid solution, mixed by ultrasonication, and stored at 4 °C for later use.

### 2.5. Preparation of the PTB Coated Electrode

The phase transition solution of the amyloid-like aggregation of BSA was prepared by mixing TCEP (50 mM, pH 5.5, pH adjusted by 5 M NaOH) and BSA (2 mg·mL^−1^) solutions at a volume ratio of 1:1. The native BSA was converted to the PTB oligomer nanoparticles and protofibrils in the solution and the PTB layer could be formed at the liquid-solid interface [23].

### 2.6. Establishment of the ECL Sensors

The SiO_2_/Mn@CeO_2_ glassy carbon electrode (GCE) was prepared by dropping 5 μL of the SiO_2_/Mn@CeO_2_ and CTS composite material on the surface of the activated electrode. After film formation, the phase transition solution was drop-cast onto the modified SiO2/Mn@CeO_2_/GCE interface and allowed to stand for several minutes to hours at room temperature. The ratio of SiO_2_, Mn@CeO_2_, and CTS was 4:4:2. The resulting PTB film formed through the aggregation of oligomer nanoparticles, which originated from protofibril aggregation in the bulk solution, at the solid–liquid interface.

### 2.7. Electrochemical Measurements

The conditions of cyclic voltammetry (CV) determination are as follows: the solution containing 5 mM K_3_[Fe(CN)_6_] and 0.1 M KCl; the scanning rate = 50 mV s^−1^; sample interval (V) = 0.001.

The conditions of electrochemical impedance spectroscopy (EIS) determination are as follows: the solution containing 10 mM K_3_[Fe(CN)_6_], K_4_[Fe(CN)_6_], and 1 M KCl; the scanning high frequency (Hz) = 10,000, low frequency (Hz) = 1, amplitude (V) = 0.005.

The conditions of ECL determination are as follows: 1×PBS buffer solution containing 30 μM luminol and 2 mM H_2_O_2_ was used as the electrolyte; the scanning range and speed were from 0 V to 0.6 V and 50 mV s^−1^; the initial scan polarity was set to positive. The voltage of the photomultiplier tube (PMT) was set to −480 V.

## 3. Results and Discussions

**Figure 1:**
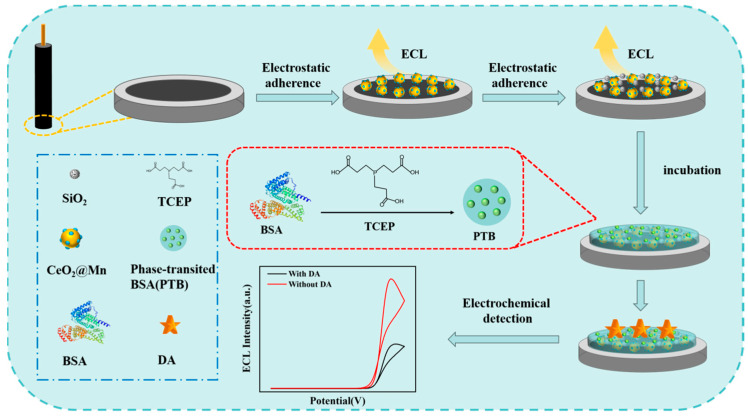
An ECL electrochemical antifouling sensor based on Mn@CeO_2_/SiO_2_ sensing was designed for the detection of DA in complex samples.

In this work, we designed a novel nanomaterial-mediated electrochemical sensor, with the principle depicted in Figure 1. Initially, based on redox reactions and metal ion doping mechanisms, manganese ions were reacted with surface oxygen atoms of CeO_2_ via a solvothermal method, enabling their incorporation into the CeO_2_ lattice to form a doped structure, thereby synthesizing Mn@CeO_2_ NPs with enzyme-like activity. Concurrently, SiO_2_ NPs possessing a high specific surface area and tunable pore sizes were synthesized by a sol-gel method, demonstrating excellent chemical stability and biocompatibility. Subsequently, a multifunctional interface was constructed on a glassy carbon electrode substrate via a chitosan-mediated composite modification strategy: the amino groups of chitosan electrostatically immobilized negatively charged Mn@CeO_2_ NPs, while its three-dimensional network structure simultaneously anchored SiO_2_ NPs, forming a composite substrate with enhanced mechanical stability and electrochemical activity. Finally, a PTB antifouling layer was prepared on the modified electrode surface via a one-step phase-transition method, reducing nonspecific adsorption through the synergistic effect of PTB surface charge balance, super hydration capacity, steric hindrance effect, and anti-adsorption properties of β-folded structures, thereby completing the construction of the electroactive sensor for detecting biological small molecules.

### 3.1. Characterization of Mn@CeO_2_ and SiO_2_

**Figure 2:**
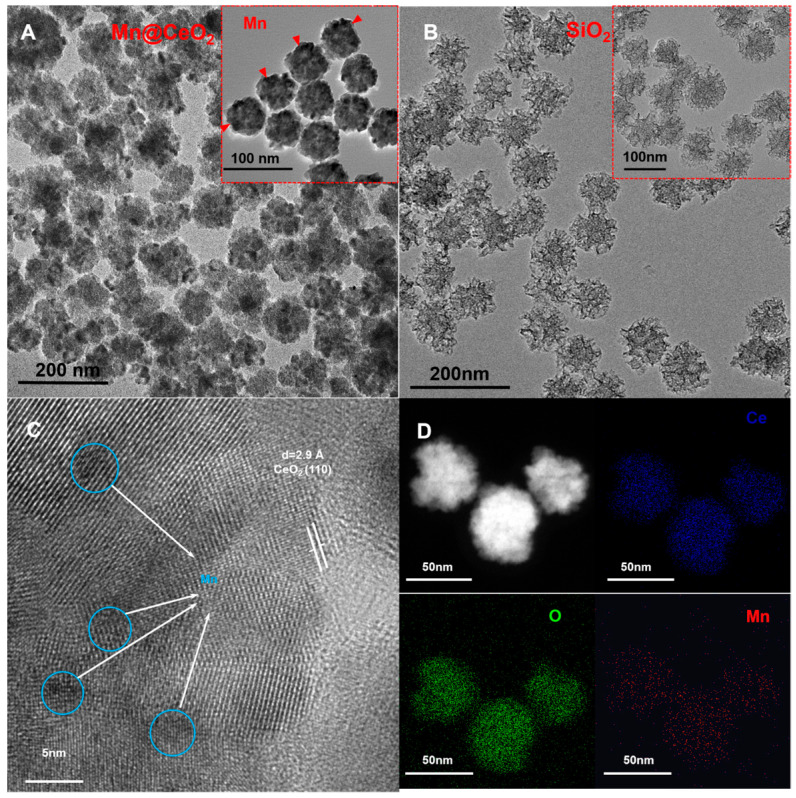
(**A**) TEM image of Mn@CeO_2_ NPs; (**B**) TEM image of SiO_2_ NPs; (**C**) high-resolution TEM image of Mn@CeO_2_ NPs; and (**D**) elemental mapping of Mn@CeO_2_ NPs.

In this study, Mn@CeO_2_ nanoparticles (NPs) were synthesized via a solvothermal method based on the approach proposed by Mohammad et al., utilizing hexamethylenetetramine (HMT) as the oxidizing agent [24]. The hydrolysis of HMT generates NH_3_^+^ and COO^−^, which provide the essential electron transfer environment for the oxidation of Ce^3+^ to Ce^4+^. The Ce^4+^ ions subsequently combine with oxygen to form the crystalline structure of CeO_2_. The incorporation of Mn^7+^ modulates the electronic structure of CeO_2_, significantly enhancing its catalytic, magnetic, and optical properties. The crystal structure, chemical composition, and morphology of the products were characterized by transmission electron microscopy (TEM) and high-resolution TEM (HR-TEM). Figure 2A reveals the morphology of Mn@CeO_2_ NP by TEM, showing highly homogeneous, near-spherical particles with rough surfaces, which, combined with the reaction yield (65%), suggests an efficient synthesis method. Based on scale bar measurements, the average particle size is determined to be 65.2 ± 3.8 nm. As shown in Figure 2B, SiO_2_ NP exhibits a uniform spherical morphology and mesoporous structure. Statistical evaluation of more than 100 particles yielded an average size of 80.0 ± 5.2 nm. High-resolution TEM (HR-TEM) analysis demonstrates that the CeO_2_ component exhibits a highly crystalline nature, with lattice fringes showing a d-spacing of 2.9 Å corresponding to the (110) plane of CeO_2_ (Figure 2C). In contrast, the Mn elements are dispersed in an amorphous form within the CeO_2_ matrix, appearing as dark spots (highlighted by blue circles). Elemental mapping results demonstrate the uniform distribution of Mn within the CeO_2_ matrix. The elemental spectra in Figure 2D further confirm the homogeneous distribution of Ce, O, and Mn elements within the Mn@CeO_2_ NPs.

**Figure 3:**
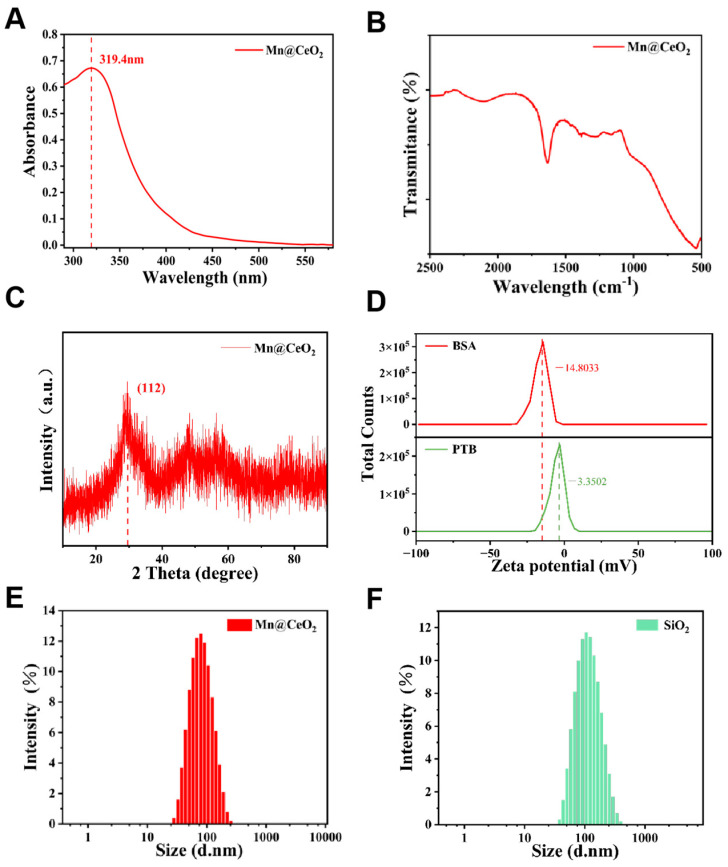
(**A**) UV–vis absorption spectra of Mn@CeO_2_; (**B**) FT–IR spectra of Mn@CeO_2_; (**C**) XRD patterns of Mn@CeO_2_; (**D**) zeta potential of BSA and PTB; (**E**) DLS of Mn@CeO_2_; (**F**) DLS of SiO_2_.

The structural and optical properties of Mn@CeO_2_ NPs were systematically analyzed using UV-Vis spectroscopy, X-ray diffraction (XRD), and Fourier transform infrared spectroscopy (FT-IR). A distinct absorption peak at 319.4 nm was observed in the UV-Vis spectrum of the aqueous dispersion of Mn@CeO_2_ NPs, exhibiting a notable blue shift compared to the characteristic absorption peak of pure CeO_2_ NPs at 328 nm [25], potentially associated with Mn doping (Figure 3A). The chemical bonding characteristics were further analyzed by FT-IR spectroscopy (Figure 3B), where characteristic peaks at 1628 cm^−1^ and 1388 cm^−1^ were attributed to the Ce-O bonds, confirming the successful synthesis of Mn@CeO_2_ NPs. According to previous studies, the CeO_2_ NPs showed significant diffraction peaks at 28.9°, 33.5°, 47.9°, and 56.8°, corresponding to the (111), (200), (220), and (311), respectively. However, the XRD analysis of Mn@CeO_2_ NPs, as depicted in Figure 3C, revealed the absence of prominent diffraction peaks except for a singular peak, which was at 29.5° corresponding to the (112) plane, indicating the amorphous nature of Mn incorporation into the CeO_2_ matrix [26].

Based on the zeta potential analysis Figure 3D, BSA exhibited a strong negative surface charge, while the PTB material displayed only a weak negative potential. This weakly anionic characteristic indicates the coexistence of both negatively and positively charged amino acid residues on the PTB surface, whose distinct charge distribution confers exceptional dye adsorption potential [27,28]. Figure 3E,F present the dynamic light scattering (DLS) size distribution profiles of Mn@CeO_2_ and SiO_2_ NPs, respectively. Both materials demonstrated monodisperse peaks with average hydrated diameters of 71.0 nm for Mn@CeO_2_ and 101.7 nm for SiO_2_. The polydispersity index (PDI) was 0.190 for Mn@CeO_2_ and 0.187 for SiO_2_. The narrow size distribution confirms excellent homogeneity of the synthesized materials, which aligns well with the well-defined morphological features observed through transmission electron microscopy (TEM) characterization.

**Figure 4:**
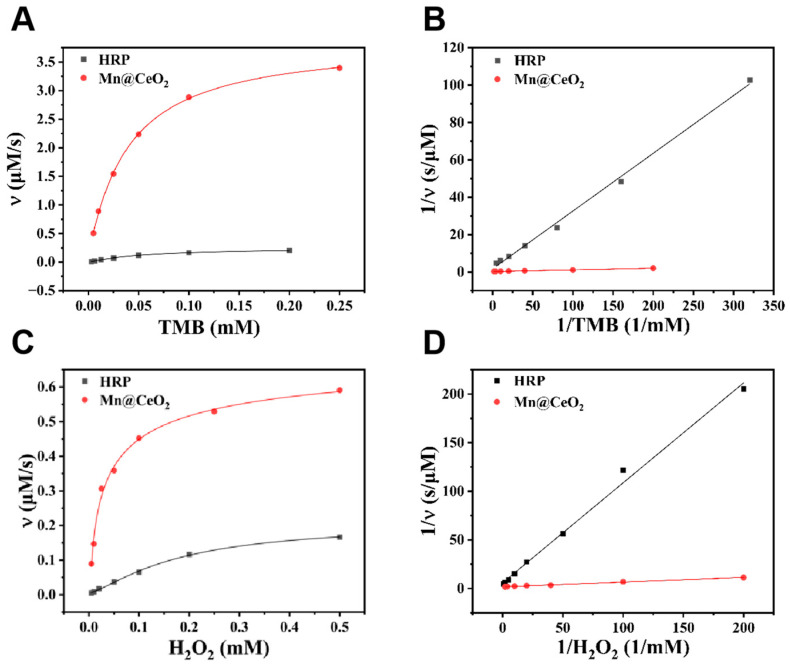
(**A**) Michaelis–Menten kinetics curves of Mn@CeO_2_ and HRP catalyzed H_2_O_2_; (**B**) Lineweaver–Burk plots of Mn@CeO_2_ and HRP catalyzed H_2_O_2_; (**C**) Michaelis–Menten kinetics curves of Mn@CeO_2_ and HRP catalyzed TMB; (**D**) Lineweaver–Burk plots of Mn@CeO_2_ and HRP catalyzed TMB.

This study systematically evaluated the enzyme-like catalytic properties of Mn@CeO_2_ NPs through steady-state kinetic analysis. Using TMB as the chromogenic substrate, the experiments determined steady-state kinetic parameters for the TMB-H_2_O_2_ reaction by maintaining fixed concentrations of one substrate. The distinct concentrations of TMB (Figure 4A,B) and H_2_O_2_ (Figure 4C,D) showed excellent agreement with both the classical Michaelis–Menten and Lineweaver–Burk models. The results revealed that Mn@CeO_2_ exhibited Michaelis–Menten constants (Km) of 0.032 mM (for TMB) and 0.029 mM (for H_2_O_2_), significantly lower than those of horseradish peroxidase (HRP), while achieving maximum reaction velocities (V_max_) of 3.74 µM/s (TMB system) and 0.60 µM/s (H_2_O_2_ system), markedly higher than HRP counterparts. These data indicate that Mn@CeO_2_ possesses superior binding affinity for both substrates compared to natural HRP, confirming its exceptional peroxidase-like activity. To visually validate catalytic performance, time-dependent chromogenic reactions (Figure S1) documented the color evolution and corresponding absorbance changes during TMB oxidation, further substantiating the rapid catalytic characteristics of Mn@CeO_2_. Furthermore, comparative analysis with reported nanozymes showed that the V_max_ values of Mn@CeO_2_ surpass those of most analogous materials (Table S1), highlighting its potential as a high-efficiency nanozyme for practical applications.

### 3.2. Electrochemical Sensing Interface Characterization

To investigate the effects of different modification materials on electrode interfacial characteristics, this study systematically characterized the electrochemical behavior of electrodes at each modification stage in 5 mM K_3_[Fe(CN)_6_] containing 0.1 M KCl using CV [29]. The GCE modified with Mn@CeO_2_ composites showed markedly increased redox peak currents (Figure S2A), resulting from enhanced electron transfer kinetics mediated by the multivalent Mn ions at the interface. When mesoporous silica and nanozymes were introduced on the electrode, the redox peak current decreased slightly, indicating that the three materials were stably fixed on the electrode surface. Notably, after PTB protein incubation, the modified electrodes showed significantly reduced peak currents, directly reflecting the successful establishment of the coating layer on the electrode surface.

EIS analysis based on Nyquist plots further elucidated the evolution of charge transfer kinetics at the electrode interface. The diameter of the semicircle in the high-frequency region directly correlates with the charge transfer resistance (Rct), which exhibits a negative correlation with electron transfer efficiency at the electrode/electrolyte interface [30,31]. In a 10 mM K_3_[Fe(CN)_6_] and K_4_[Fe(CN)_6_] solution containing 1 M KCl, the composite electrodes demonstrated distinct electron transfer capabilities at different modification stages. As shown in Figure S2B, compared to the bare GCE, Mn@CeO_2_/GCE and SiO_2_/Mn@CeO_2_/GCE showed markedly reduced Rct, attributed to the excellent electron transfer capabilities of the nanomaterials. In contrast, PTB/SiO_2_/Mn@CeO_2_/GCE displayed increased Rct, confirming the successful immobilization of the anti-fouling coating on the electrode surface. These EIS results are consistent with the CV data in Figure S2A.

To enhance the detection performance, a series of optimization experiments were carried out. As shown in Figure S3A, the luminol-H_2_O_2_ luminescence system was optimized by fixing the luminol concentration at 30 μM while varying the H_2_O_2_ concentration. Mn@CeO_2_ exhibited the highest catalytic efficiency when the H_2_O_2_ concentration reached 2 mM. Subsequently, the Mn@CeO_2_ loading concentration on the electrode surface was optimized, revealing peak catalytic performance at 1 μg/mL (Figure S3B). This optimal concentration likely represents a balance between insufficient catalytic activity at lower loadings and compromised electron transfer efficiency at higher loadings. Further optimization of the reaction conditions demonstrated optimal ECL response at pH 10 (Figure S3C) and showed temperature-dependent behavior, with signal intensity increasing from 20 °C to 40 °C (Figure S3D). Notably, the system maintained high detection sensitivity even at 70 °C, highlighting the exceptional thermal stability of Mn@CeO_2_, which is attributed to its robust crystalline structure that preserves catalytic activity under harsh conditions.

**Figure 5:**
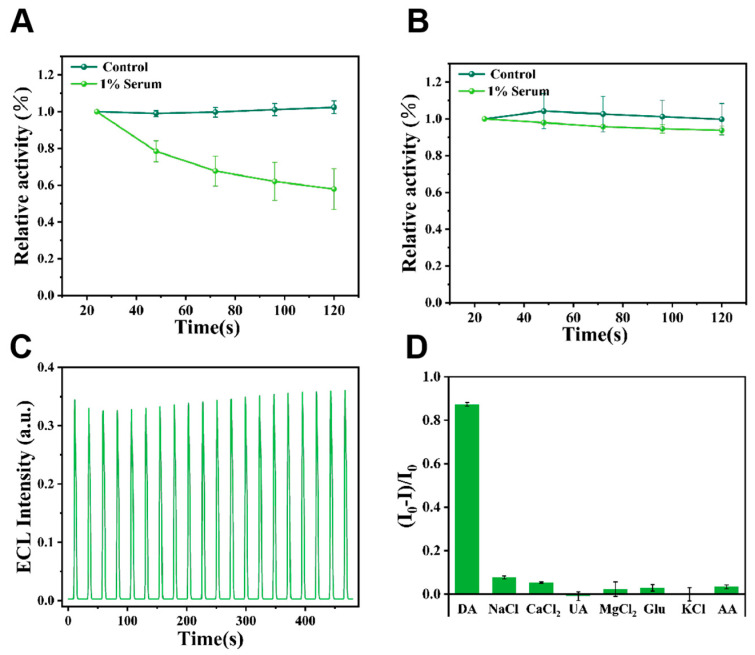
Analytical performance of the ECL sensor. (**A**) Interference immunity of Mn@CeO_2_/GCE within 5 cycles; (**B**) interference immunity of PTB/Mn@CeO_2_/GCE within 5 cycles; (**C**) stability test within 20 cycles; (**D**) specificity against various types of interferences.

The anti-interference capability of the PTB-modified sensor was assessed. As shown in Figure 5A,B, the PTB/Mn@CeO_2_/GCE demonstrated significantly higher anti-interference performance compared to Mn@CeO_2_/GCE. In the presence of 1% FBS, the ECL signal of the Mn@CeO_2_/GCE sensor decreased markedly, dropping to 56.6% of its initial value after 120 s. In contrast, the PTB/Mn@CeO_2_/GCE sensor exhibited exceptional stability, retaining 94.0% of its initial signal after 120 s.

To evaluate stability, the modified electrode (PTB/Mn@CeO_2_/GCE) was subjected to 20 consecutive potential scans in 1× PBS electrolyte containing 2 mM H_2_O_2_ and 30 μM luminol. As shown in Figure 5C, the sensor produced well-defined and stable ECL signals with a relative standard deviation (RSD) of 3.35%, confirming its robust operational stability.

Specificity is a critical criterion for assessing sensor performance. The selectivity of the sensor was tested against various analogs and interfering substances, including ions (K^+^, Na^+^, Ca^2+^, Mg^2+^, Cl^−^), as well as biomolecules such as uric acid (UA), glucose (Glu), and ascorbic acid (AA), which are known to potentially interfere with detection. Results in Figure 5D revealed that only (DA) caused significant quenching of the ECL signal. This indicates that common biomolecules or inorganic ions had no notable impact on the conductive activity of the sensor, demonstrating its excellent selectivity for DA detection.

**Figure 6:**
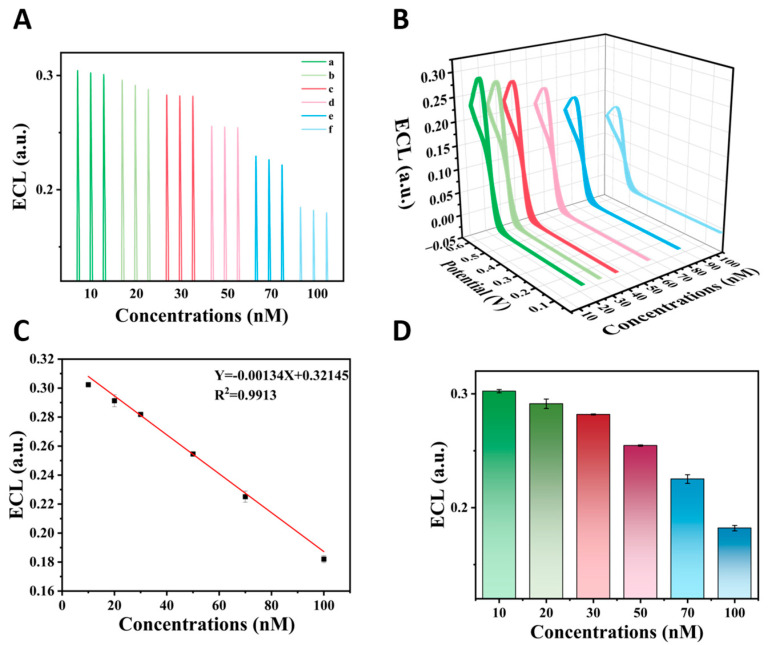
Analytical performance of the ECL sensor. ECL intensity (**A**,**B**,**D**) and calibration curve (**C**) for DA at different concentrations: (a) 10 nM, (b) 20 nM, (c) 30 nM, (d) 50 nM, (e) 70 nM, and (f) 100 nM.

To further assess the biosensing performance, the relationship between DA concentration and ECL signal was investigated under optimized conditions. Concentration-dependent variations in ECL signal intensity were observed across different DA levels (Figure 6A,B,D). Quantitative analysis demonstrated a linear calibration relationship (Figure 6C) with the regression equation y = −0.00134X + 0.32145 (R^2^ = 0.9913), showing excellent linearity in the 10–100 nM concentration range. The limit of detection (LOD) was calculated as 6.61 nM (*n* ≥ 5) using the 3δ/|b| criterion (where δ represents the standard deviation of blank measurements and |b| denotes the absolute slope value), indicating high sensitivity for DA quantification. This detection performance was superior to previously reported DA assays (Table S2), demonstrating the enhanced analytical capabilities of the sensing platform.

To validate the practical applicability of the system, DA detection was performed in samples containing 1% FBS. For DA at varying concentrations, the sensor demonstrated recovery rates within the range of 101–103%, Table S3. These results confirm that the PTB/Mn@CeO_2_/GCE electrochemical biosensor is highly effective for detecting DA in serum samples, showcasing its potential for clinical and diagnostic applications.

## 4. Conclusions

This study successfully developed a nanozyme-based ECL biosensor for the detection of biomolecules in serum. The results demonstrated that the Mn@CeO_2_ nanocomposite exhibited peroxidase-like activity significantly higher than that of horseradish peroxidase (HRP). The introduction of PTB coating on the sensing interface effectively suppressed the nonspecific adsorption of interferences from serum samples, maintaining the detection performance above 94%. Under optimal conditions, the sensor showed a good linear response to DA with a detection limit as low as 6.61 nM. Therefore, the developed PTB/SiO_2_/Mn@CeO_2_/GCE ECL biosensor not only possesses high sensitivity and a low detection limit but also exhibits excellent anti-interference capability and stability for DA detection in complex biological samples. This approach provides a new strategy for the development of high-performance ECL biosensors and holds great potential for applications in clinical diagnostics and biomedical research.

## Data Availability

Data is contained within the article. Data will be made available on request.

## Supplementary Materials

The following supporting information can be downloaded at: https://www.mdpi.com/article/10.3390/bios15070411/s1, Figure S1: TMB color development experiment with different concentrations of Mn@CeO_2_; Figure S2: CV and EIS characterization of different modified electrodes; Figure S3: Conditional optimization of sensing performance; Table S1: Main apparatus used in this experiment; Table S2: Comparison of kinetic parameters of CeO_2_-based nanozymes with reported peroxidase analogues; Table S3: Recovery of DA in 1000-diluted serum sample; Table S4: Comparison of the analytical performance of the established nanosensing-based DA detection method. Refs. [32,33,34,35,36,37,38,39,40,41,42,43,44,45,46,47] cited in Supplementary Materials.
