# Supplementary material for: Mn-Doped CeO2 Nanozyme-Integrated Mesoporous Interfaces for High-Sensitivity Antifouling Electrochemiluminescence Biosensing
Source: Biosensors (Basel). 2025 Jun 27;15(7):411. doi: 10.3390/bios15070411 (PMC12293221; doi:10.3390/bios15070411)
Supplement: Supplementary file 1 [file biosensors-15-00411-s001.zip › biosensors-3701824-supplementary.pdf]

Mn-Doped CeO_2 Nanozyme-Integrated Mesoporous Interfaces for High-Sensitivity Antifouling Electrochemiluminescence Biosensing

Guanze Huang [1], Haiyan Qiu [1], Huiping Chen [1], Wanxuan Li [1], Yufei Zhang [1], Minfang Huang [3], Tingting Zhang [2,*], Xiaoxin Xu [1,*] and Shanwen Hu [1]

[1] Department of Health Inspection and Quarantine, School of Public Health, Fujian Medical University, Fuzhou 350122, China; hgz000506@fjmu.edu.cn (G.H.); 2220210169@fjmu.edu.cn (H.Q.); chp2240210259@fjmu.edu.cn (H.C.); liwanxuan@stu.fjmu.edu.cn (W.L.); zhangyufei@stu.fjmu.edu.cn (Y.Z.); shanwenhu@fjmu.edu.cn (S.H.)

[2] Jiangsu Key Laboratory for Chemistry of Low-Dimensional Materials, School of Chemistry and Chemical Engineering, Huaiyin Normal University, Huaian 223300, China

[3] Key Laboratory for Analytical Science of Food Safety and Biology, Ministry of Education, College of Chemistry, Fuzhou University, Fuzhou 350002, China; 221320071@fzu.edu.cn

* Correspondence: tingtingzhang@hytc.edu.cn (T.Z.); xuxiaoxin139976@fjmu.edu.cn (X.X.)

Table S1. Main apparatus used in this experiment

Figure S1. TMB color development experiment with different concentrations of Mn@CeO_2

Figure S2. CV and EIS characterization of different modified electrodes

Figure S3. Conditional optimization of sensing performance

Table S2. Comparison of kinetic parameters of CeO_2-based nanozymes with reported peroxidase analogues

Table S3. Recovery of DA in 1000-diluted serum sample

Table S4. Comparison of the analytical performance of the established nanosensing-based DA detection method

Table S1. Main apparatus used in this experiment.

**Apparatus**	**Specification/Model**	**Manufacturer**
Pure water/ultrapure water integrated system	Milli-Q Integarl	Millipore
Constant temperature metal bath	ThermoStat plus	Eppendorf
Transmission electron microscopy	JEM-2100	JEOL
High-resolution TEM and HAADF-STEM	JEOL JEM-F200	JEOL
X-ray diffraction (XRD)	Rigaku D/max 2500	Rigaku
Zetasizer	ZEN series	Malvern Instrument Company Ltd
Electrochemical workstation	CHI660E	Shanghai Chenhua Instrument Company Ltd
Refrigerated high-speed centrifuge	H1850R	CENCE
Constant temperature heating magnetic stirrer	DF-101D	YUHUA
Dark box ultraviolet analyzer	ZF-20D	YUHUA
Freeze dryer	FD-1A-50	BIOCOOL

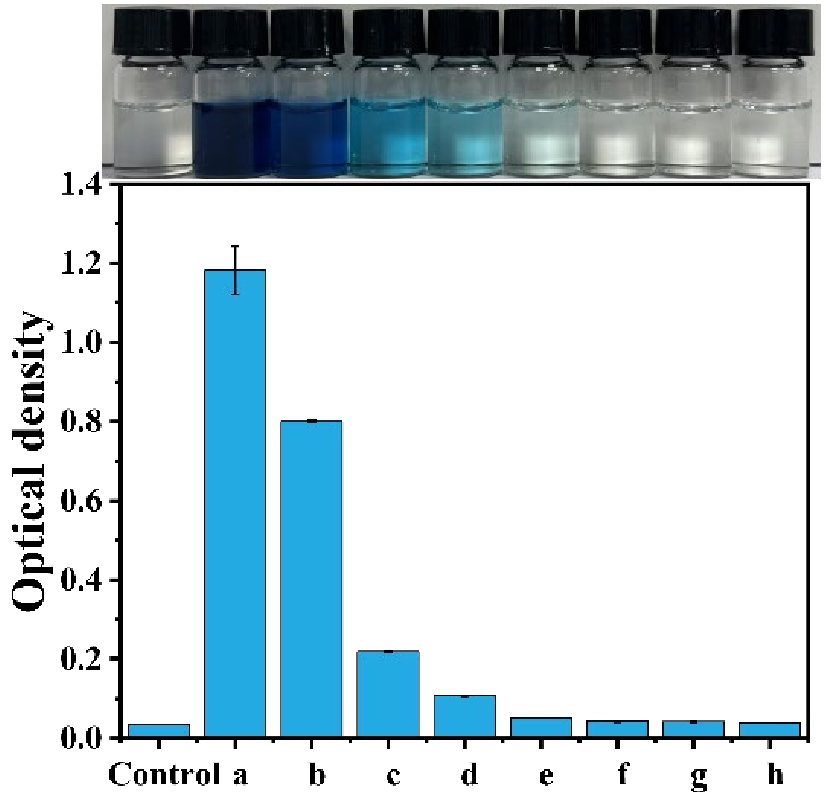

Figure S1. Visualization and absorbance of 3,3',5,5'-tetramethylbenzidine chromogenic catalyzed by different concentrations of Mn@CeO_2. Concentration changes: (a) 50 μg; (b) 25 μg; (c) 5 μg; (d) 2.5 μg; (e) 500 ng; (f) 50 ng; (g) 5 ng; (h) 500 pg.

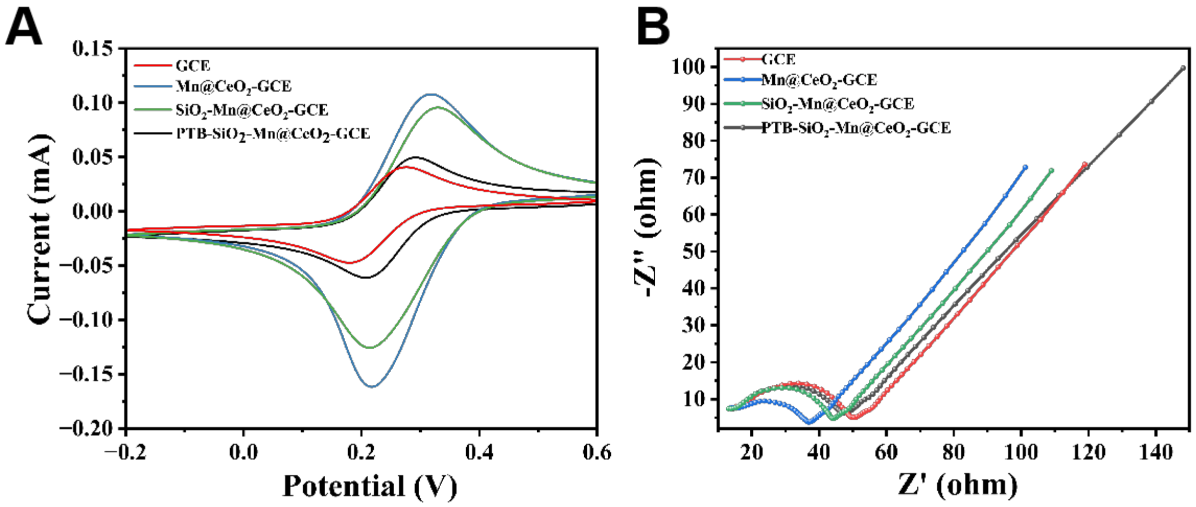

Figure S2. (A) The cyclic voltammetry curves in 5 mM $K_3[Fe(CN)_6]$ containing 0.1 M KCl, and (B) The Electrochemical impedance spectroscopy data in 10 mM $K_3[Fe(CN)_6]$ and $K_4[Fe(CN)_6]$ solution containing 1 M KCl of GCE, Mn@CeO_2/GCE, SiO_2/Mn@CeO_2/GCE, PTB/SiO_2/Mn@CeO_2/GCE.

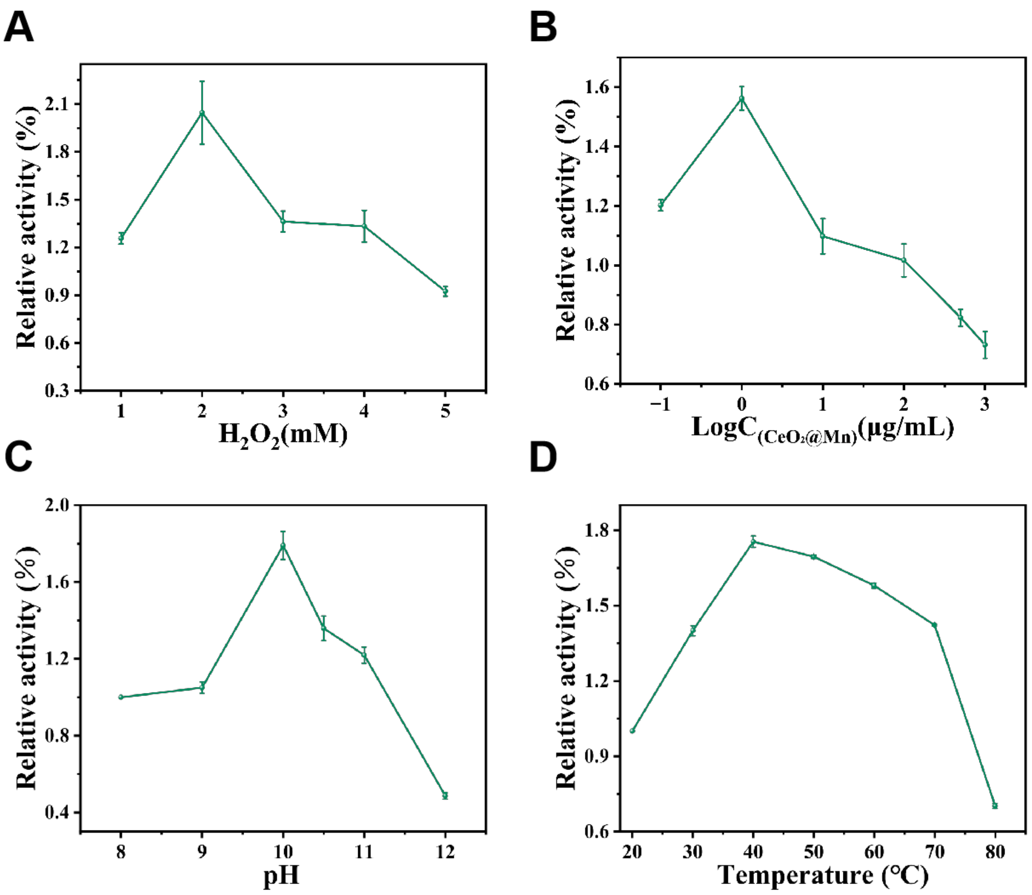

Figure S3. Effects of (A) Concentration of H_2O_2, (B) Concentration of Mn@CeO_2, (C) pH, (D) Temperature.

Table S2. Comparison of kinetic parameters of CeO_2-based nanozymes with reported peroxidase analogues.

**Material**	**Substrate**	**Km (mM)**	**Vmax (μM/s)**	**Ref.**
FeS_2/SiO_2	TMB	0.948	0.310	[1]
	H_2O_2	0.013	0.181	
CeO_2/Co_3O_4@NCH	TMB	0.086	0.161	[2]
	H_2O_2	4.001	0.071	
Co_3O_4@CeO_2	TMB	0.140	0.413	[3]
	H_2O_2	7.090	0.433	
CeO_2/CuO@CC	TMB	0.027	0.076	[4]
CeO_2@ LDH	H_2O_2	18.630	2.730	[5]
PdNPs/TCNF	TMB	0.055	0.101	[6]
	H_2O_2	0.039	0.076	
AuAg-rGO	TMB	0.484	0.043	[7]
	H_2O_2	0.248	0.088	
HRP	TMB	0.182	0.589	This work
	H_2O_2	0.180	0.174	
Mn/CeO_2NPs	TMB	0.032	3.740	This work
	H_2O_2	0.029	0.600	

Table S3 Recovery of DA in 1000-diluted serum sample

**Sample**	**Added Concentration (nM)**	**Measured Concentration (nM)**	**Average concentration (nM)**	**Recovery (%)**
1	20	18.8; 20.8; 22.1	20.6	103
2	50	46.5; 48.9; 50.6	20.6	103
3	100	97.0; 99.0; 106.7	100.9	101

Table S4 Comparison of the analytical performance of the established nanosensing-based DA detection method.

Method	Sensor	Linear range (nM)	LOD (nM)	Ref.
Colorimetric	ATP/$CoFe_2O_4$@ZIF-8@ZIF-6	2.0×10^2 ~ 1.8×10^4	400	[8]
Colorimetric	Fe_3O_4@C@AgNPs	5.0×10^2 ~ 8.0×10^4	120	[9]
Colorimetric	MoS_2-CPBNPs	1.0×10^3 ~ 1.0×10^5	90	[10]
Colorimetric	Van-Pt_2NPs	1.0×10^4 ~ 7.0×10^5	854	[11]
SERRS	AgNPs@PVP	1.0×10^2 ~ 1.0×10^4	40	[12]
Electrochemical	Co@NCNTs/NC/GCE	3.0×10^1 ~ 7.1×10^5	9	[13]
Electrochemical	rGO-$Cu_3H_2Mo_2O_{10}$	1.0×10^3 ~ 1.0×10^4 and 3.4×10^4 ~ 1.25×10^5	158	[14]
Electrochemical	TiO_2/Ti_3C_2	1.0×10^3 ~1.0×10^6	520	[15]
Electrochemical	CeO_2/Co_3O_4	1.3×10^2 ~6.0×10^5	130	[16]
Electrochemical	PTB/SiO_2/CeO_2@Mn	1.0×10^1 ~ 1.0×10^2	5.96	This work

References

1. Huang, X.; Xia, F.; Nan, Z. Fabrication of FeS_2/SiO_2 Double Mesoporous Hollow Spheres as an Artificial Peroxidase and Rapid Determination of H_2O_2 and Glutathione. ACS Appl Mater Interfaces. 2020, 12(41), 46539-46548.

2. Cao, X.; Zhao, S.; Liu, X.; Zhu, X.; Gao, Y.; Liu, Q. CeO_2/Co_3O_4@N-doped hollow carbon microspheres with improved peroxidase-like activity for the determination of quercetin. Anal Bioanal Chem. 2022, 414(16), 4767-4775.

3. Jampaiah, D.; Srinivasa, R.T.; Coyle, V.E.; Nafady, A., Bhargava, S.K. Co_3O_4@CeO_2 hybrid flower-like microspheres: a strong synergistic peroxidase-mimicking artificial enzyme with high sensitivity for glucose detection. J Mater Chem B. 2017, 5(4), 720-730.

4. Hao,J.; Feng, J.; Sun, S.; Cao, Z.; Xu, W.; Hu, L.; Yao, W.; Yan, Z. Reliable ratiometric colorimetric monitoring of dopamine in practice based on the catalytic signal amplification of nano CeO_2/CuO modified carboxylated chitosan. Chem. Eng. Sci. 2024, 295, 120193.

5. Chen, F.; Ma, X.; Cao, X.; Dou, Y.; Guan, S.; Qiu, X.; Han, J. An effective antioxidant to mitigate reperfusion injury by tailoring CeO_2 electronic structure on layered double hydroxide nanosheets. Chem. Eng. J. 2023, 475, 146190.

6. Dadigala, R.; Bandi, R.; Han, S.Y.; Kwon, G.J.; Lee, S.H. Rapid in-situ growth of enzyme-mimicking Pd nanoparticles on TEMPO-oxidized nanocellulose for the efficient detection of ascorbic acid. Int J Biol Macromol. 2023, 234, 123657.

7. Das, D.P.; Boruah, P.K.; Sarmah, P.; Dutta, R.; Boukherroub, D.R.; Das, D.M.R. A Facile Preparation of Reduced Graphene Oxide Capped AuAg Bimetallic Nanoparticles: A Selective Nanozyme for Glutathione Detection, Chemistryselect 2022, 7(40), e202203415.

8. Esmaeili, A.; Dehghan, G.; Dadakhani, S.; Fathinia, M.; Haghighat, H.; Khataee, A. Development of ATP-modified $CoFe_2O_4$@ZIF-8@ZIF-67 core-shell nanozyme for sensitive colorimetric detection of dopamine. Microchemical Journal 2024, 204, 111027.

9. Alula, M.T.; Hendricks-Leukes, N.R. Silver nanoparticles loaded carbon-magnetic nanocomposites: A nanozyme for colorimetric detection of dopamine. Spectrochim Acta A Mol Biomol Spectrosc. 2024, 322, 124830.

10. Zhu, Z.; Gong, L.; Miao, X.; Chen, C.; Su, S. Prussian Blue Nanoparticle Supported MoS_2 Nanocomposites as a Peroxidase-Like Nanozyme for Colorimetric Sensing of Dopamine. Biosensors 2022, 12(5), 260.

11. Xue, Y.; Liu, K.; Gao, M.; Zhang, T.; Wang, L.; Cui, Y.; Ji, X.; Ma, G.; Hu, J. Vancomycin-Stabilized Platinum Nanoparticles with Oxidase-like Activity for Sensitive Dopamine Detection. Biomolecules 2023, 13(9), 1312.

12. Zhao, L.; Du, X.; Xu, G.; Song, P. Nanozyme catalyzed-SERRS sensor for the recognition of dopamine based on AgNPs@PVP with oxidase-like activity. Spectrochim Acta A. 2024, 307, 123606.

13. Lu, N.; Yan, X.; Gu, Y.; Zhang, T.; Liu, Y.; Song, Y.; Xu, Z.; Xing, Y.; Li, X.; Zhang, Z.; Zhai, S. Cobalt-decorated 3D hybrid nanozyme: A catalytic amplification platform with intrinsic oxidase-like activity. Electrochim. Acta. 2021, 395, 139197.

14 Singh, G.; Kushwaha, A.; Sharma, M. Electrochemistry of rGO-$Cu_3H_2Mo_2O_{10}$ cuboidal nanostructures: An effective detection of neurotransmitter dopamine in blood serum sample. J. Electroanal. Chem. 2021, 880, 114889.

15. Chen, Y.; Li, X.; Cai, G.; Li, M.; Tang, D. In situ formation of (001)TiO_2/Ti_3C_2 heterojunctions for enhanced photoelectrochemical detection of dopamine. Electrochem. Commun. 2021, 125, 106987.

16. Y.X. Ding, X.L. Zhao, P. Wu, R.R. Wang, L.L. Xie, Z.H. Li, Z.G. Zhu, H.L. Zhao, M.B. Lan, ZIF-67 MOF derived Co-Based CeO_2 electrochemical sensor for dopamine, Electrochimica Acta. 2023, 463, 142802.
